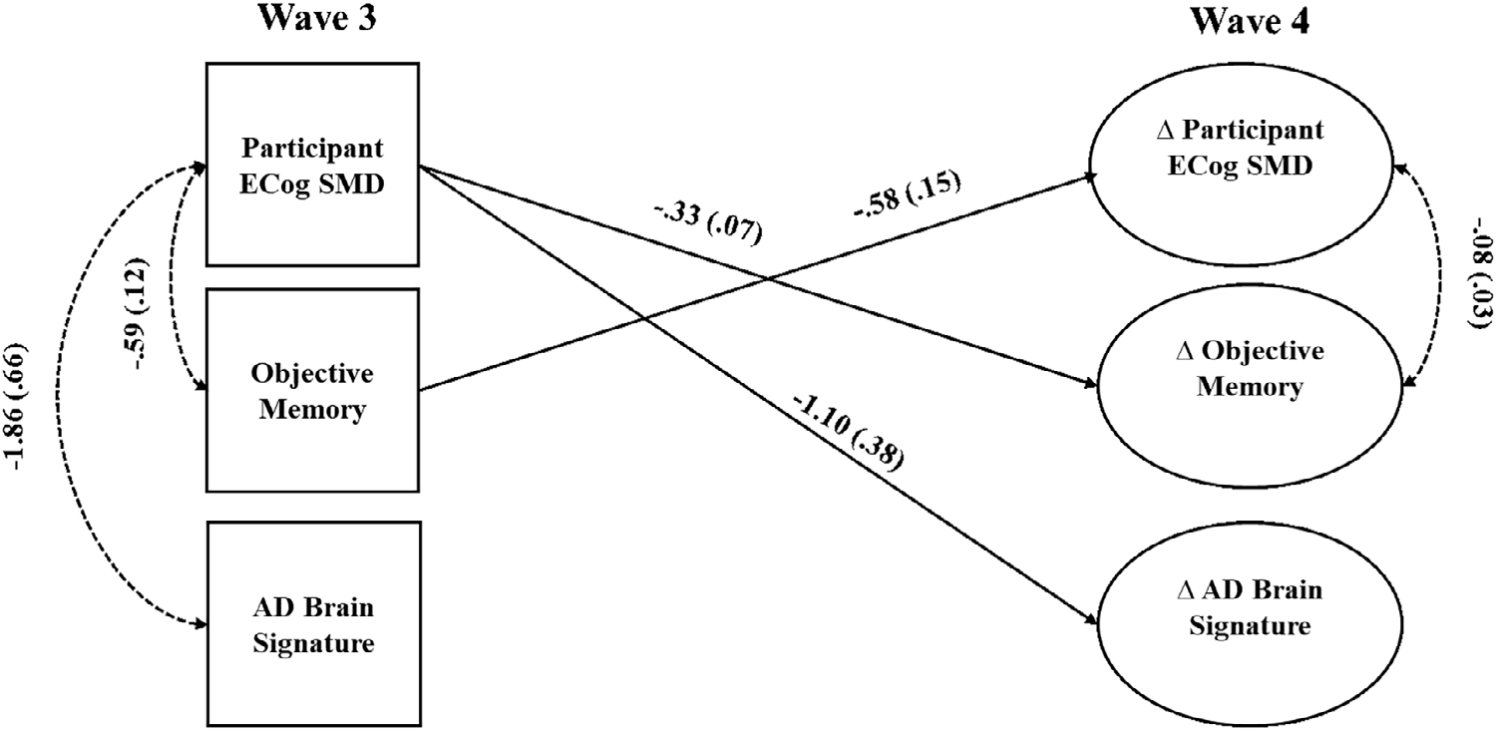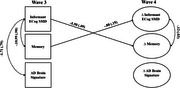# Association of Subjective Memory Decline with Objective Memory Decline and Alzheimer’s Related Brain Structure

**DOI:** 10.1002/alz70862_109863

**Published:** 2025-12-23

**Authors:** Tyler R. Bell, Daniel E. Gustavson, Carol E. Franz, Matthew S. Panizzon, Christine Fennema‐Notestine, Jeremy A. Elman, Rosemary Toomey, Amy J. Jak, Diane M. Jacobs, Anders M. Dale, Rongxiang Tang, William S. Kremen

**Affiliations:** ^1^ University of California San Diego, La Jolla, CA USA; ^2^ Center for Behavior Genetics of Aging, University of California, San Diego, La Jolla, CA USA; ^3^ University of Colorado Boulder, Boulder, CO USA; ^4^ University of California, San Diego, La Jolla, CA USA; ^5^ Boston University, Boston, MA USA; ^6^ VA San Diego Healthcare System, San Diego, CA USA; ^7^ Department of Neurosciences, University of California San Diego, La Jolla, CA USA; ^8^ UCSD Shiley‐Marcos Alzheimer's Disease Research Center, La Jolla, CA USA; ^9^ Alzheimer’s Disease Cooperative Study, University of California San Diego, La Jolla, CA USA; ^10^ Department of Cognitive Science, University of California, San Diego, La Jolla, CA USA; ^11^ Center for Multimodal Imaging and Genetics, University of California, San Diego, La Jolla, CA USA; ^12^ Department of Neurosciences, University of California, San Diego, La Jolla, CA USA; ^13^ Texas A&M University, College Station, TX USA

## Abstract

**Background:**

Subjective memory decline (SMD) is a putative early indicator of Alzheimer’s disease (AD). However, its association with changes in objective memory and AD‐related brain structure is mixed, which may be partly due to reliance on single‐item measures of subjective decline. The Everyday Cognition scale (ECog) was developed to improve detection of early AD risk by leveraging multiple items, focusing on behaviors rather than general perceptions, and including informant ratings.

**Method:**

Data were collected from cognitively unimpaired men at waves 3 and 4 (mean ages 68 and 74) of the Vietnam Era Twin Study of Aging (VETSA). The ECog memory subscale, assessing SMD over 10 years, was completed by participants (wave 3: *n* = 904; wave 4: *n* = 516) and informants (wave 3: *n* = 811; wave 4: *n* = 473). Participants also completed objective memory testing, summarized as latent factor scores. For a subset completing MRI (wave 3: *n* = 388; wave 4: *n* = 191), AD brain signature scores were derived from hippocampal volume and cortical thickness in seven AD‐related regions.

Structural equation models estimated associations of ECog‐SMD with objective memory and AD brain signature scores at wave 3, and predictive associations between ECog‐SMD at wave 3 and changes in objective memory and AD brain signature scores by wave 4. Associations between changes in ECog‐SMD and changes in objective memory and AD brain signature scores were also examined. Covariates included age 20 cognitive ability, depressive symptoms, and state anxiety.

**Result:**

Shown in Figures 1 and 2, ECog‐SMD at wave 3 was associated with worse objective memory (participant: b=‐.59, *p* < .001; informant: b=10.99, *p* = .001) and lower AD brain signature scores (participant: b=‐1.86, *p* = .007; informant: b=‐1.71, *p* = .018). ECog‐SMD also predicted declines in objective memory (participant: b=‐.33, *p* < .001; informant: ‐5.50, ps<.001) and reductions in AD brain signature (participant :b=‐1.10, *p* < .001) by wave 4. At wave 4, increases in ECog‐SMD correlated with declines in objective memory (participant: b=‐.08, *p* = .007; informant: b=‐.12, *p* = .013) but were not associated with changes in AD brain signatures (participant and informant: ps>.05).

**Conclusion:**

Participant and informant ECog ratings of SMD reflected concurrent differences in objective memory decline and AD‐related neurodegeneration. Participant‐reported SMD were associated with future AD‐related neurodegeneration, potentially capturing risk undetected by neuropsychological testing or informant reports.